# Identification of 4-Amino-Thieno[2,3-*d*]Pyrimidines as QcrB Inhibitors in Mycobacterium tuberculosis

**DOI:** 10.1128/mSphere.00606-19

**Published:** 2019-09-11

**Authors:** Gregory A. Harrison, Anne E. Mayer Bridwell, Megh Singh, Keshav Jayaraman, Leslie A. Weiss, Rachel L. Kinsella, Janessa S. Aneke, Kelly Flentie, Miranda E. Schene, Margaret Gaggioli, Samantha D. Solomon, Scott A. Wildman, Marvin J. Meyers, Christina L. Stallings

**Affiliations:** a Department of Molecular Microbiology, Washington University School of Medicine, Saint Louis, Missouri, USA; b Center for World Health and Medicine, Saint Louis University School of Medicine, Saint Louis, Missouri, USA; c University of Wisconsin Carbone Cancer Center, School of Medicine and Public Health, University of Wisconsin—Madison, Madison, Wisconsin, USA; d Department of Chemistry, Saint Louis University, Saint Louis, Missouri, USA; University of Rochester

**Keywords:** CydAB, *Mycobacterium tuberculosis*, QcrB, antibiotic, cytochrome, drug discovery, respiration

## Abstract

Antibiotic resistance is a global crisis that threatens our ability to treat bacterial infections, such as tuberculosis, caused by Mycobacterium tuberculosis. Of the 10 million cases of tuberculosis in 2017, approximately 19% of new cases and 43% of previously treated cases were caused by strains of M. tuberculosis resistant to at least one frontline antibiotic. There is a clear need for new therapies that target these genetically resistant strains. Here, we report the discovery of a new series of antimycobacterial compounds, 4-amino-thieno[2,3-*d*]pyrimidines, that potently inhibit the growth of M. tuberculosis. To elucidate the mechanism by which these compounds inhibit M. tuberculosis, we selected for mutants resistant to a representative 4-amino-thieno[2,3-*d*]pyrimidine and sequenced these strains to identify the mutations that confer resistance. We isolated a total of 12 resistant mutants, each of which harbored a nonsynonymous mutation in the gene *qcrB*, which encodes a subunit of the electron transport chain (ETC) enzyme cytochrome *bc*_1_ oxidoreductase, leading us to hypothesize that 4-amino-thieno[2,3-*d*]pyrimidines target this enzyme complex. We found that addition of 4-amino-thieno[2,3-*d*]pyrimidines to M. tuberculosis cultures resulted in a decrease in ATP levels, supporting our model that these compounds inhibit the M. tuberculosis ETC. Furthermore, 4-amino-thieno[2,3-*d*]pyrimidines had enhanced activity against a mutant of M. tuberculosis deficient in cytochrome *bd* oxidase, which is a hallmark of cytochrome *bc*_1_ inhibitors. Therefore, 4-amino-thieno[2,3-*d*]pyrimidines represent a novel series of QcrB inhibitors that build on the growing number of chemical scaffolds that are able to inhibit the mycobacterial cytochrome *bc*_1_ complex.

**IMPORTANCE** The global tuberculosis (TB) epidemic has been exacerbated by the rise in drug-resistant TB cases worldwide. To tackle this crisis, it is necessary to identify new vulnerable drug targets in Mycobacterium tuberculosis, the causative agent of TB, and develop compounds that can inhibit the bacterium through novel mechanisms of action. The QcrB subunit of the electron transport chain enzyme cytochrome *bc*_1_ has recently been validated to be a potential drug target. In the current work, we report the discovery of a new class of QcrB inhibitors, 4-amino-thieno[2,3-*d*]pyrimidines, that potently inhibit M. tuberculosis growth *in vitro*. These compounds are chemically distinct from previously reported QcrB inhibitors, and therefore, 4-amino-thieno[2,3-*d*]pyrimidines represent a new scaffold that can be exploited to inhibit this drug target.

## Abstract (precis)

The global tuberculosis (TB) epidemic has been exacerbated by the rise in drug-resistant TB cases worldwide. To tackle this crisis, it is necessary to identify new vulnerable drug targets in Mycobacterium tuberculosis, the causative agent of TB, and develop compounds that can inhibit the bacterium through novel mechanisms of action. The QcrB subunit of the electron transport chain enzyme cytochrome *bc*_1_ has recently been validated to be a potential drug target. In the current work, we report the discovery of a new class of QcrB inhibitors, 4-amino-thieno[2,3-*d*]pyrimidines, that potently inhibit M. tuberculosis growth *in vitro*. These compounds are chemically distinct from previously reported QcrB inhibitors, and therefore, 4-amino-thieno[2,3-*d*]pyrimidines represent a new scaffold that can be exploited to inhibit this drug target.

## INTRODUCTION

Infection with Mycobacterium tuberculosis resulted in over 9 million new cases of tuberculosis (TB) and 1.5 million deaths in 2017, making it the most deadly infectious agent in the world (1). This epidemic is exacerbated by the rise of multidrug-resistant (MDR) TB cases that are resistant to at least the two frontline antibiotics used to treat TB, isoniazid and rifampin. MDR-TB constituted 3.6% of new TB cases in 2017 and 17% of previously treated TB cases, with rates of MDR-TB being as high as 50% among previously treated TB cases in some countries (1). Furthermore, 8.5% of MDR-TB cases in 2017 were estimated to be extensively drug resistant (XDR), which are also resistant to a fluoroquinolone and a second-line injectable drug (1). This rise in drug resistance and the scarcity of drugs in the pipeline have made it clear that we are not equipped to successfully battle the ongoing TB epidemic.

In 2012, the diarylquinoline compound bedaquiline (Sirturo), which inhibits the mycobacterial ATP synthase (2), was approved to treat MDR-TB patients (3). The success of this new anti-TB antibiotic fueled interest in mycobacterial energy metabolism pathways as vulnerable targets for new antibiotic development. More recently, the imidazopyridine amide (IPA) Q203 (telacebec) was identified to be a potent antimycobacterial compound that targets QcrB, a subunit of the mycobacterial cytochrome *bc*_1_:*aa*_3_ oxidoreductase in the electron transport chain (ETC) (4). Q203 is currently in phase II clinical trials for the treatment of TB (5). Since the discovery of Q203 and additional imidazopyridine amides (4, 6–10), a number of compounds have been identified that are also reported to target QcrB, including pyrazolo[1,5-*a*]pyridine-3-carboxamides (11–13), imidazo[2,1-*b*]thiazole-5-carboxamides (14), pyrrolo[3,4-*c*]pyridine-1,3(2H)-diones (15), lansoprazole sulfide (16), 2-(quinolin-4-yloxy)acetamides (17, 18), arylvinylpiperazine amides (19), phenoxyalkylbenzimidazoles (20–22), and morpholino thiophenes (23).

Here, we present the discovery of 4-amino-thieno[2,3-*d*]pyrimidines as a new series of QcrB inhibitors that potently inhibit M. tuberculosis growth and that are chemically distinct from previously identified QcrB inhibitors. This work adds to the growing number of QcrB inhibitors that have recently been identified and contributes to our understanding of ways to exploit this target in the development of new chemotherapeutic strategies for TB treatment.

## RESULTS

### Identification of 4-amino-thieno[2,3-*d*]pyrimidines with growth-inhibitory activity in Mycobacterium smegmatis and M. tuberculosis.

**FIG 1:**
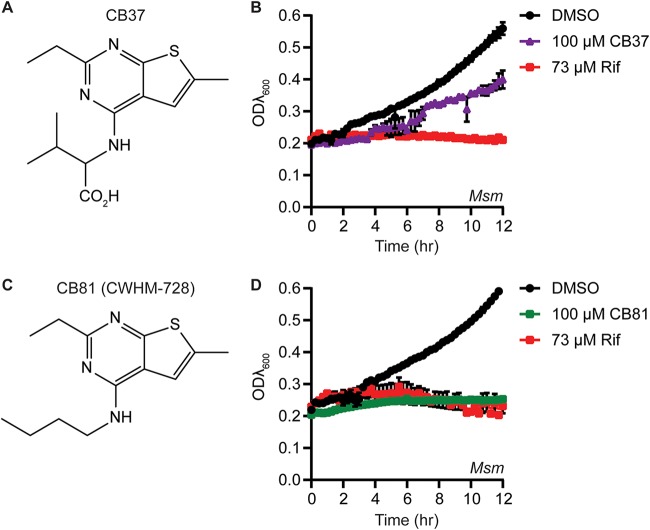
CB37 and CB81 are 4-amino-thieno[2,3-*d*]pyrimidines that inhibit the growth of M. smegmatis. (A) Structure of CB37. (B) M. smegmatis (*Msm*) strain csm208 (Table 2) was incubated in LB medium in the presence of DMSO, 100 μM CB37, or 73 μM rifampin (Rif), and the optical density at 600 nm (ODλ_600_) was measured periodically over the course of 12 h in a plate reader (*n* = 2 replicates). (C) Structure of CB81, which was resynthesized and renamed CWHM-728. (D) M. smegmatis strain csm208 was incubated in LB medium in the presence of DMSO, 100 μM CB81, or 73 μM rifampin, and the ODλ_600_ was measured over the course of 12 h in a plate reader (*n* = 3 replicates).

In an effort to identify novel inhibitors of mycobacteria, we screened a selection of 78 small-molecule nucleotide mimetics purchased from ChemBridge Corporation for compounds that inhibit the growth of M. smegmatis in a high-throughput liquid culture assay. From these screens, we identified a 4-amino-thieno[2,3-*d*]pyrimidine (CB37) that inhibited the growth of M. smegmatis (Fig. 1A and B). We hypothesized that the charged carboxylate group on CB37 may greatly reduce penetration through the cell envelope of the mycobacteria and selected a set of 9 structurally related compounds that did not contain the carboxylate group but that contained the 2-ethyl-6-methylthieno[2,3-*d*]pyrimidine core (nearest neighbors) to purchase and assay for inhibition of M. smegmatis (Fig. 1; see also Fig. S1 and entries 2 to 10 in Table S1 in the supplemental material). Eight of the compounds showed either similar levels of growth inhibition against M. smegmatis as CB37 or no growth inhibition at all (Fig. S1). However, one of these compounds, CB81, showed improved growth inhibition in M. smegmatis (Fig. 1C and D). We resynthesized CB81 and henceforth designate it CWHM-728.

10.1128/mSphere.00606-19.2 FIG S1 Related 4-amino-thieno[2,3-*d*]pyrimidines do not inhibit the growth of M. smegmatis more than CB37 does. M. smegmatis strain csm208 was incubated in the presence of the indicated 4-amino-thieno[2,3-*d*]pyrimidine at 100 μM, and the optical density (OD) was measured periodically over the course of 12 h in a plate reader (*n* = 3 experiments). (A to F) DMSO and 73 μM rifampin were included as controls. The control data for panels A to F are the same in each panel because all the compounds were tested in the same assay plate. (G and H) DMSO and 1.1 mM streptomycin were included as controls. The control data for panels G and H are the same in both panels because these compounds were tested in the same assay plate. Download FIG S1, TIF file, 1.3 MB. Copyright © 2019 Harrison et al. 2019 Harrison et al. This content is distributed under the terms of the Creative Commons Attribution 4.0 International license.

**FIG 2:**
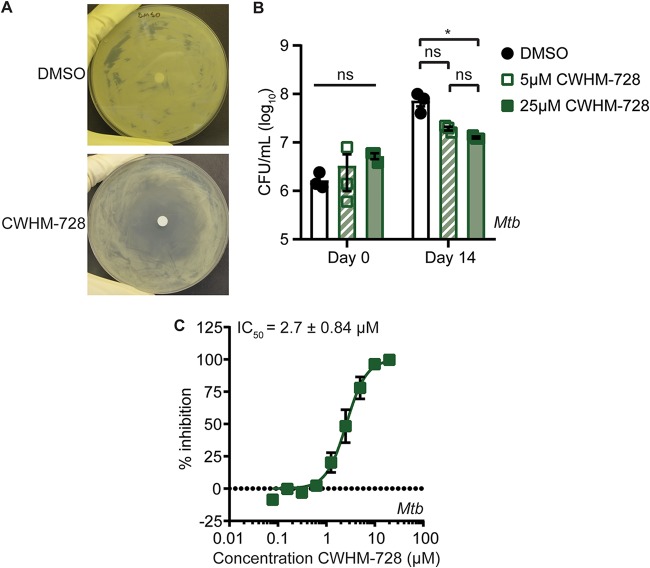
CWHM-728 is a 4-amino-thieno[2,3-*d*]pyrimidine compound that exhibits anti-M. tuberculosis activity. (A) A zone-of-inhibition assay was performed by spreading 2.5 × 10^8^ CFU of M. tuberculosis on a plate containing 7H11 agar medium, placing a sterile disk in the center, and pipetting 5 μl of 100% DMSO or 100 mM CWHM-728 on the disk. The plate was incubated at 37°C for 10 days. Representative images from at least 3 independent experiments are shown. (B) M. tuberculosis (*Mtb*) was incubated in 7H9 liquid medium in the presence of DMSO or CWHM-728 at the indicated concentrations. At the indicated time points, samples were collected and plated onto 7H11 agar medium containing no antibiotics to enumerate the CFU per milliliter (*n* = 3 replicates). Statistical comparisons for the number of CFU per milliliter on the final day of the treatment are depicted. *P* values were determined by two-way analysis of variance with Tukey’s posttest. *, *P* < 0.05; ns, not significant. (C) M. tuberculosis was incubated with increasing concentrations of CWHM-728, and bacterial respiration and metabolism were measured using the MABA (*n* = 3 replicates). The best-fit curve and IC_50_ values ± standard deviation were calculated using GraphPad Prism software.

10.1128/mSphere.00606-19.6 TABLE S1 Structure-activity relationship studies around CB37. *^a^*clogP, calculated log(partition coefficient, P). Values were calculated using the CDD Vault platform. *^b^*Values are from Fig. 2C. Download Table S1, DOCX file, 0.1 MB. Copyright © 2019 Harrison et al. 2019 Harrison et al. This content is distributed under the terms of the Creative Commons Attribution 4.0 International license.

To determine if CWHM-728 also had activity against M. tuberculosis, we performed zone-of-inhibition assays with the wild-type (WT) M. tuberculosis Erdman strain by spreading approximately 2.5 × 10^8^ CFU of bacteria on an agar plate and spotting 5 μl of a 100 mM stock of CWHM-728 dissolved in dimethyl sulfoxide (DMSO) onto a disk in the center of the plate. After incubation at 37°C for 10 days, the bacteria formed a lawn and a zone absent of bacterial growth indicated growth inhibition by the compound. DMSO had no effect on M. tuberculosis growth in this assay and did not generate a zone of clearing on its own, whereas incubation of M. tuberculosis with CWHM-728 resulted in growth inhibition (Fig. 2A). To test if CWHM-728 has a bacteriostatic or a bactericidal effect on M. tuberculosis, we cultured M. tuberculosis in liquid medium in the presence of DMSO or 5 μM or 25 μM CWHM-728 and enumerated the viable CFU after 14 days of incubation (Fig. 2B). While exposure to 5 μM CWHM-728 for 14 days caused a slight but not statistically significant decrease in the number of CFU compared to that for the DMSO-treated control, exposure to 25 μM CWHM-728 caused a significant reduction in the number of CFU per milliliter compared to that for the DMSO-treated control. However, exposure to CWHM-728 did not decrease the number of viable CFU below the number of initial CFU on day 0, indicating that over 14 days, this concentration of CWHM-728 had a bacteriostatic effect on M. tuberculosis.

To identify the half-maximal inhibitory concentration (IC_50_) of CWHM-728 against M. tuberculosis, we used a microplate alamarBlue assay (MABA), which is a high-throughput assay commonly used to evaluate the efficacy of antimycobacterial compounds (24). The MABA utilizes the redox-sensitive dye resazurin, which is blue in its oxidized form but becomes reduced to the pink fluorescent compound resorufin as a result of bacterial metabolism and respiration. Fluorescence can therefore be quantified as a readout for M. tuberculosis metabolism and respiration, which serves as a proxy for M. tuberculosis growth and/or survival. We incubated M. tuberculosis in the presence of increasing concentrations of CWHM-728 and found that CWHM-728 inhibited M. tuberculosis in the MABA with an IC_50_ of 2.7 ± 0.84 μM (Fig. 2C).

To explore chemical modifications that would improve upon the growth-inhibitory activity of CWHM-728 and develop structure-activity relationships (SAR), we used the MABA to test if the analogs that we had previously obtained in which the *n*-butyl side chain was replaced with other aliphatic groups had inhibitory activity against M. tuberculosis (see entries 1 to 10 in Table S1). Analogs that had side chains with ionizable groups (entries 1 and 3) and polar groups, such as hydroxyl (entry 4), had poor activity, with IC_50_ values being greater than 25 μM. Capping the hydroxyl group as methoxy (entries 5 and 6) restored some potency, and isopropoxy (entry 7) yielded a compound 8-fold more potent than the compound with a butyl side chain (entry 2). Cyclopropyl was tolerated (entry 8), with a submicromolar IC_50_, while slightly bulkier cyclopentyl and piperidine side chains resulted in very poor potency (entries 9 and 10).

To further extend the SAR, we synthesized analogs of CWHM-728 containing lipophilic side chains, and the resulting analogs were evaluated in the MABA (Table S2). Replacing the *n*-butyl side chain with *t*-butyl caused a nearly 10-fold decrease in potency (entry 2), but trifluoro-*n*-butyl and isopentyl variants (entries 3 and 4) were found to be greater than 10-fold more potent than the original *n*-butyl (entry 1). Capping the NH with a methyl group (entry 5) demonstrated little effect on potency. Finally, a series of phenyl and alkylphenyl side chains was prepared and showed remarkable SAR, with potency being strongly dependent on chain length (entries 6 to 9).

**FIG 3:**
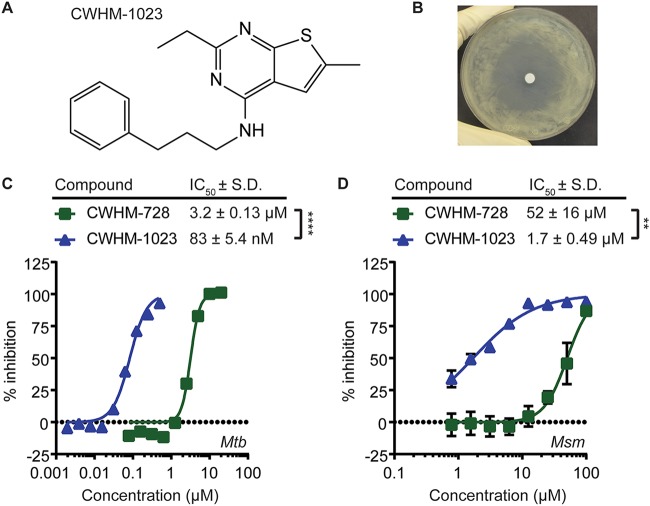
CWHM-1023 is a 4-amino-thieno[2,3-*d*]pyrimidine with enhanced activity against mycobacteria. (A) Chemical structure of CWHM-1023. (B) Zone-of-inhibition assay with CHWM-1023. M. tuberculosis (2.5 × 10^8^ CFU) was spread on a plate containing 7H11 agar medium, a sterile disk was placed in the center, and 5 μl of 100 mM CWHM-1023 was pipetted onto the disk. The plate was incubated at 37°C for 10 days. A representative image from at least 2 independent experiments is shown. (C and D) M. tuberculosis (*Mtb*) (C) or M. smegmatis (*Msm*) (D) was incubated in the presence of increasing concentrations of either CWHM-728 or CWHM-1023, before bacterial respiration and metabolism were measured using the MABA (*n* = 3 replicates). Best-fit curves and IC_50_ values were calculated using GraphPad Prism software. *P* values were determined by Student's *t* test. **, *P* < 0.01; ****, *P* < 0.0001.

10.1128/mSphere.00606-19.7 TABLE S2 Structure-activity relationship studies around CWHM-728 and comparison to known M. tuberculosis inhibitors. *^a^*clogP, calculated log(partition coefficient, P). Values were calculated using the CDD Vault platform. *^b^*Values are from Fig. 3C. Download Table S2, DOCX file, 0.1 MB. Copyright © 2019 Harrison et al. 2019 Harrison et al. This content is distributed under the terms of the Creative Commons Attribution 4.0 International license.

The most potent compound from our SAR evaluation was CWHM-1023, which shared the 4-amino-thieno[2,3-*d*]pyrimidine core scaffold with CWHM-728 but which contained a 3-phenylpropyl side chain (Fig. 3A). We found that CWHM-1023 inhibited M. tuberculosis growth in the zone-of-inhibition assay (Fig. 3B) and inhibited M. tuberculosis in the MABA with an IC_50_ of 83 ± 5.4 nM (Fig. 3C). This is a 38-fold improvement in potency compared to that of CWHM-728, which had an IC_50_ of 3.2 ± 0.13 μM in this experiment (Fig. 3C). Thus, CWHM-728 and CWHM-1023 are 4-amino-thieno[2,3-*d*]pyrimidines that inhibit M. tuberculosis at low micromolar and submicromolar concentrations, respectively. Following this same trend in potency, we found that the IC_50_s of CWHM-728 and CWHM-1023 for M. smegmatis in the MABA were 52 ± 16 μM and 1.7 ± 0.49 μM, respectively (Fig. 3D).

### Mutations in *qcrB* confer resistance to 4-amino-thieno[2,3-*d*]pyrimidines.

To identify the target of the 4-amino-thieno[2,3-*d*]pyrimidines, we selected for M. tuberculosis mutants that were resistant to CWHM-728 by plating approximately 2.5 × 10^8^ CFU of WT M. tuberculosis Erdman on 7H11 agar medium containing 10 μM CWHM-728 and incubating the culture at 37°C for 12 weeks. We found that spontaneous CWHM-728-resistant mutant colonies emerged at an approximate frequency of 1 in 2.3 × 10^7^. To identify the genetic basis for CWHM-728 resistance, we performed whole-genome sequencing on four CWHM-728-resistant strains and found that all four strains harbored missense mutations in the *qcrB* gene. Three of the strains, isolated from a single culture, had mutations resulting in an A178T amino acid change, and one strain, isolated from a second independent culture, had a mutation generating a V338G substitution (Table 1). We then sequenced the *qcrB* locus in 8 additional CWHM-728-resistant mutants isolated from a third independent culture and identified a missense mutation in *qcrB* in every isolate (Table 1). One strain harbored the A178T mutation, and 3 more had the V338G mutation that we had isolated previously. In addition, we found that the remaining isolates harbored a A178V, G175S, or G315S mutation in *qcrB*.

**TABLE 1:** CWHM-728-resistant isolates harbor mutations in *qcrB*^*a*^

Culture no. and resistant strain	Mutation in *qcrB*
Culture 1, CB81R	V338G^*b*^
Culture 2	
D3	A178T^*b*^
81.2	A178T^*b*^
81.5	A178T^*b*^
Culture 3	
CB81R a	A178V
CB81R b	G175S
CB81R c	V338G
CB81R d	V338G
CB81R f	A178T
CB81R g	A178V
CB81R i	G315S
CB81R j	V338G

a The CWHM-728-resistant strains isolated in this study are listed with the mutation identified in *qcrB*.

b The mutations were identified by whole-genome sequencing. All other mutations were identified by Sanger sequencing of the *qcrB* locus.

**FIG 4:**
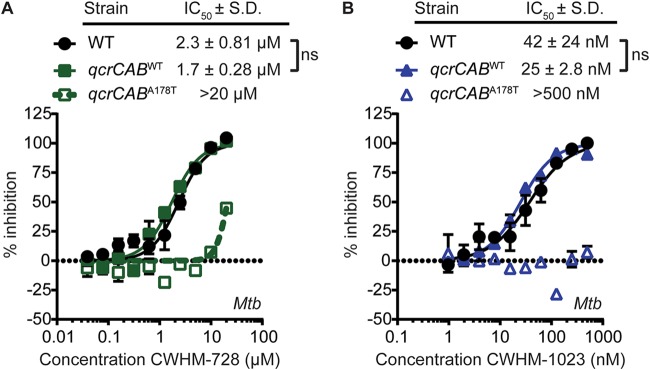
A mutation in *qcrB* confers resistance to 4-amino-thieno[2,3-*d*]pyrimidines. The M. tuberculosis (*Mtb*) WT or the M. tuberculosis Δ*qcrCAB* mutant complemented with either *qcrCAB*^WT^ or *qcrCAB*^A178T^ was incubated in the presence of increasing concentrations of CWHM-728 (A) or CWHM-1023 (B), and then bacterial respiration and metabolism were measured using the MABA (*n* = 3). Best-fit curves and IC_50_ values ± standard deviation (S.D.) were calculated using GraphPad Prism software. ns, not significant by Student's *t* test.

The *qcrB* gene is located within an operon containing *ctaE*, *qcrC*, and *qcrA*. The *qcrCAB* gene cassette encodes all 3 subunits of the cytochrome *bc*_1_ complex (25). This complex is localized to the plasma membrane and forms a supercomplex with the *aa*_3_-type cytochrome oxidase encoded by *ctaBCDE* (26, 27). The resulting cytochrome *bc*_1_:*aa*_3_ oxidase complex catalyzes the terminal electron transfer reaction in the mycobacterial ETC, transferring electrons from menaquinol, the lipid electron carrier in the membrane, to the terminal electron acceptor oxygen. We sought to confirm that mutations in *qcrB* were sufficient to confer resistance to 4-amino-thieno[2,3-*d*]pyrimidines to rule out the possibility that the CWHM-728-resistant mutants harbored mutations elsewhere in the genome that contributed to resistance. For these studies, we engineered M. tuberculosis strains that expressed either WT *qcrCAB* (*qcrCAB*^WT^) or *qcrCAB* with the A178T substitution (*qcrCAB*^A178T^) from the chromosomal *attB* site and deleted the endogenous *qcrCAB* locus. We then monitored the activity of CWHM-728 (Fig. 4A) and CWHM-1023 (Fig. 4B) against these strains using the MABA. We found that the strain expressing *qcrCAB*^A178T^ exhibited reduced sensitivity to both compounds compared to that of WT M. tuberculosis or the isogenic *qcrCAB*^WT^ control strain, confirming that this single amino acid change in *qcrB* confers resistance to 4-amino-thieno[2,3-*d*]pyrimidines. These data are consistent with a model in which the 4-amino-thieno[2,3-*d*]pyrimidines target cytochrome *bc*_1_.

**FIG 5:**
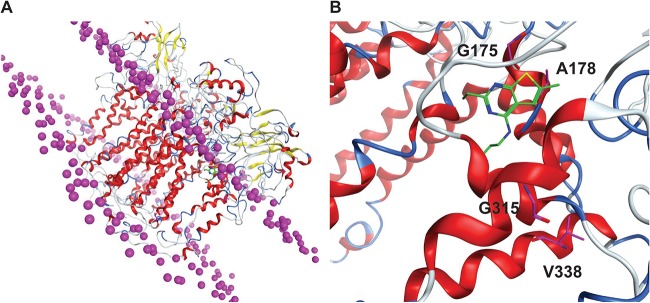
Molecular modeling of CWHM-728 in the predicted structure of M. tuberculosis QcrB. (A) QcrB homology model embedded in the lipid bilayer, with the lipid head group phosphorus being shown as magenta spheres. (B) QcrB binding site shown with CHWM-728 docked (green). The residues mutated in resistant mutants are highlighted in magenta.

Based on our finding that mutations in the *qcrB* gene confer resistance to 4-amino-thieno[2,3-*d*]pyrimidines, we propose that this new class of antimycobacterial compounds targets QcrB. To better understand how mutations in *qcrB* confer resistance to 4-amino-thieno[2,3-*d*]pyrimidines, we used computational modeling to predict the structure of the M. tuberculosis QcrB protein based on a previously published structure for QcrB from Rhodobacter sphaeroides, which shares 16% amino acid identity with the M. tuberculosis QcrB (28) (Fig. 5A). We docked CWHM-728 onto the predicted structure of QcrB and found that it localized near the putative menaquinol binding site of QcrB. Based on our computational model, we speculate that the A178T, A178V, or G175S mutation in QcrB would disrupt the contacts required for 4-amino-thieno[2,3-*d*]pyrimidines to bind QcrB (Fig. 5B). The V338 and G315 residues are not located where they would directly interact with CWHM-728 but are positioned on neighboring helices within a very tightly packed region, such that changes in amino acid identity could possibly affect the position of those helices and disrupt the 4-amino-thieno[2,3-*d*]pyrimidine binding site.

The predicted binding site for the 4-amino-thieno[2,3-*d*]pyrimidines is similar to what has previously been reported from similar modeling studies for the QcrB inhibitor Q203 (29). We investigated whether the 4-amino-thieno[2,3-*d*]pyrimidine-resistant strain expressing *qcrCAB*^A178T^ was more resistant to Q203 than the isogenic *qcrCAB*^WT^ control strain and found that the *qcrB*^A178T^ mutation did not affect Q203 sensitivity in the MABA (Fig. S2). These data suggest that while the *qcrB*^A178T^ allele is sufficient to confer resistance to 4-amino-thieno[2,3-*d*]pyrimidines, it does not confer cross-resistance to Q203, possibly because the A187T mutation is not sufficient to disrupt the binding of Q203 to QcrB.

10.1128/mSphere.00606-19.3 FIG S2 M. tuberculosis harboring the *qcrB*^A178T^ allele is not cross-resistant to Q203. WT or M. tuberculosis (*Mtb*) Δ*qcrCAB* complemented with either *qcrCAB*^WT^ or *qcrCAB*^A178T^ was incubated in the presence of increasing concentrations of Q203, and then bacterial respiration and metabolism were measured using the MABA (*n* = 3 experiments). Best-fit curves and IC_50_ values ± standard deviation (S.D.) were calculated using GraphPad Prism software. *P* values were determined by one-way analysis of variance with Tukey’s posttest. *, *P* < 0.05; **, *P* < 0.01; ns, not significant. Download FIG S2, TIF file, 0.8 MB. Copyright © 2019 Harrison et al. 2019 Harrison et al. This content is distributed under the terms of the Creative Commons Attribution 4.0 International license.

### 4-Amino-thieno[2,3-*d*]pyrimidines deplete ATP levels in M. smegmatis and M. tuberculosis.

**FIG 6:**
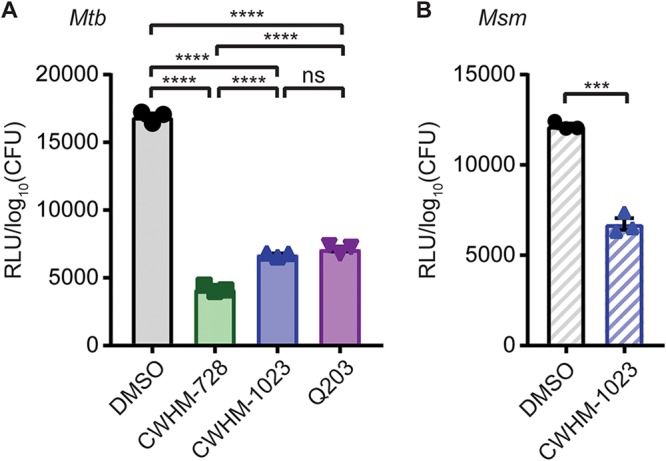
4-Amino-thieno[2,3-*d*]pyrimidines deplete mycobacterial ATP levels. (A) M. tuberculosis (*Mtb*) was incubated in the presence of 1 μM CWHM-728, 1 μM CWHM-1023, or 400 nM Q203 for 24 h, the samples were heat inactivated, and ATP was quantified using the BacTiter-Glo assay (*n* = 3). (B) M. smegmatis (*Msm*) was incubated in the presence of 10 μM CWHM-1023 for 12 h, and ATP was quantified using the BacTiter-Glo assay (*n* = 3). In both panels, ATP levels were normalized to the number of log_10_ CFU to account for differences in bacterial numbers due to differences in growth. *P* values were determined by one-way analysis of variance with Tukey’s posttest (A) or Student's *t* test (B). ***, *P* < 0.001; ****, *P* < 0.0001; ns, not significant.

Based on our model that 4-amino-thieno[2,3-*d*]pyrimidines target the QcrB subunit of cytochrome *bc*_1_, we hypothesized that CWHM-728 and CWHM-1023 inhibit the mycobacterial ETC. To test this hypothesis, we incubated M. tuberculosis in the presence of 1 μM CWHM-728, 1 μM CWHM-1023, or 400 nM Q203 for 24 h and measured the ATP levels in the bacteria using the BacTiter-Glo assay (Promega). We found that treatment with CWHM-728, CWHM-1023, and Q203 decreased the ATP levels in M. tuberculosis by 75%, 60%, and 58%, respectively (Fig. 6A). Similar results were observed in M. smegmatis, where 10 μM CWHM-1023 caused a 41% decrease in ATP levels in M. smegmatis (Fig. 6B). These data support the possibility that 4-amino-thieno[2,3-*d*]pyrimidines target a complex involved in energy generation, consistent with our hypothesis that these compounds inhibit QcrB in the mycobacterial ETC.

### M. smegmatis and M. tuberculosis lacking cytochrome *bd* are hypersensitive to 4-amino-thieno[2,3-*d*]pyrimidines.

**FIG 7:**
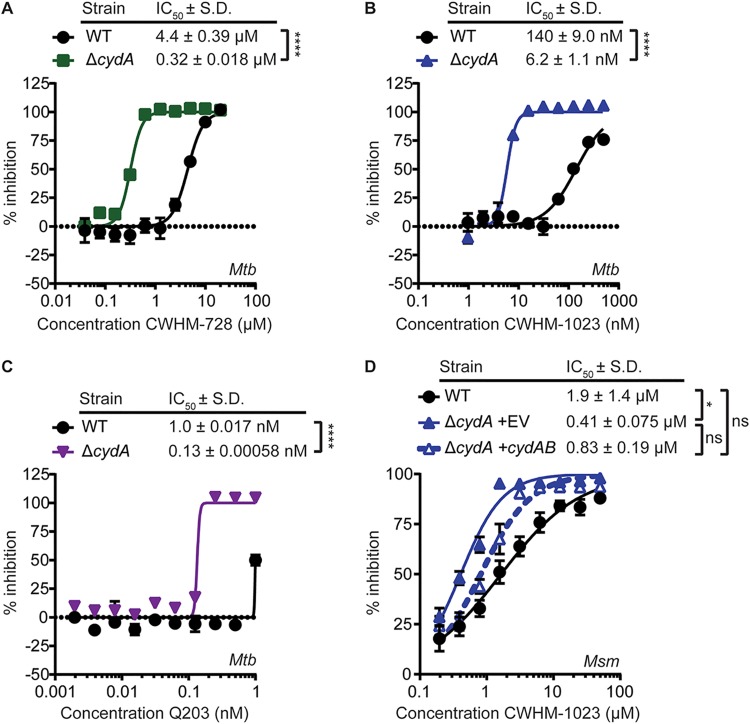
Mycobacteria lacking cytochrome *bd* have increased sensitivity to 4-amino-thieno[2,3-*d*]pyrimidines. (A to C) Either WT M. tuberculosis (*Mtb*) or M. tuberculosis Δ*cydA* was incubated in the presence of increasing concentrations of CWHM-728 (A), CWHM-1023 (B), or Q203 (C), and respiration and metabolism were measured using the MABA (*n* = 3 replicates). (D) WT M. smegmatis (*Msm*) or M. smegmatis Δ*cydA* complemented with the empty vector (EV) or with a plasmid harboring *cydAB* was incubated in the presence of increasing concentrations of CWHM-1023, and respiration and metabolism were measured using the MABA (*n* = 3 replicates). (A to D) Best-fit curves and IC_50_ values ± standard deviation (S.D.) were generated using GraphPad Prism software. *P* values were determined by Student's *t* test (A to C) or one-way analysis of variance with Tukey’s posttest (D). *, *P* < 0.05; ****, *P* < 0.0001; ns, not significant.

Mycobacteria have a branched ETC that can terminate either in the cytochrome *bc*_1_:*aa*_3_ terminal oxidase or in the cytochrome *bd* terminal oxidase, encoded by *cydAB* (25, 30). Cytochrome *bc*_1_:*aa*_3_ oxidase and cytochrome *bd* oxidase have somewhat overlapping roles in the ETC to transfer electrons from menaquinol to oxygen, where the cytochrome *bd* oxidase can partially compensate for the loss of cytochrome *bc*_1_:*aa*_3_ oxidase activity, as evidenced by the observation that mutants of cytochrome *bd* exhibit increased sensitivity to cytochrome *bc*_1_ inhibitors (8, 31). Therefore, we hypothesized that 4-amino-thieno[2,3-*d*]pyrimidines would exhibit enhanced activity against mycobacteria lacking cytochrome *bd* compared with that of the WT strains. To test this hypothesis, we generated M. tuberculosis Δ*cydA* and M. smegmatis Δ*cydA* mutants, which lack cytochrome *bd*, and measured the sensitivity of these mutant strains to 4-amino-thieno[2,3-*d*]pyrimidines compared to that of the WT strains. We found that deletion of *cydA* increased the sensitivity of M. tuberculosis to CWHM-728 13.8-fold (Fig. 7A), CWHM-1023 22.6-fold (Fig. 7B), and Q203 7.7-fold (Fig. 7C), as measured by a decrease in the MABA IC_50_ compared to that for WT M. tuberculosis. Additionally, we found that CWHM-1023 had a 4.6-fold lower IC_50_ against M. smegmatis Δ*cydA* than against WT M. smegmatis, which was partially complemented by expressing *cydAB* in this strain (Fig. 7D). These data demonstrate that genetic deletion of cytochrome *bd* sensitizes mycobacteria to inhibition by 4-amino-thieno[2,3-*d*]pyrimidines, which is consistent with our hypothesis that these compounds target the QcrB subunit of cytochrome *bc*_1_.

## DISCUSSION

There is a dire need for new anti-TB drugs that shorten treatment regimens and that are effective against MDR and XDR strains of M. tuberculosis. To begin to address this need, two anti-TB agents have recently received accelerated regulatory approval from the U.S. Food and Drug Administration (FDA) (3). These two drugs, bedaquiline (Sirturo) and delamanid, are the first antibiotics to be approved for the treatment of TB in the last 50 years (32). The discovery and development of bedaquiline in particular have raised general interest in targeting M. tuberculosis ETC and respiration as a therapeutic strategy.

Simultaneously, numerous phenotypic screens have identified QcrB inhibitors that inhibit the growth of M. tuberculosis in axenic culture and/or in macrophages (4, 8, 15, 16, 19, 21, 23, 33), raising further interest in the ETC as a drug target. The most clinically advanced of these QcrB inhibitors is the imidazopyridine amide (IPA) Q203 (telacebec), which is currently in clinical trials for the treatment of TB (5). In this study, we report the discovery of a novel class of QcrB inhibitors, 4-amino-thieno[2,3-*d*]pyrimidines, that are chemically distinct from the previously identified QcrB inhibitors. Therefore, the new chemical scaffold described here adds to the growing chemical space that can be exploited to target the mycobacterial cytochrome *bc*_1_ complex. The most potent 4-amino-thieno[2,3-*d*]pyrimidine inhibitor of QcrB that we identified, CWHM-1023, had an IC_50_ of approximately 83 nM for M. tuberculosis in the MABA assay (see Table S2 in the supplemental material). This is in comparison with the IC_50_s for the known respiration inhibitors Q203, bedaquiline, and thioridazine, which are 1.5 nM, <78 nM, and 11.2 μM, respectively, against M. tuberculosis in the MABA (Table S2).

Mycobacteria have a branched ETC, whereby electrons can be shuttled either to cytochrome *bc*_1_ or to cytochrome *bd* to be transferred to the terminal electron acceptor, O_2_ (30). Cytochrome *bc*_1_ is proposed to be bioenergetically more efficient than cytochrome *bd* under standard aerobic conditions (25). However, cytochrome *bd* is induced under conditions of low oxygen and contributes to mycobacterial fitness under microaerobic conditions, suggesting that cytochrome *bd* can support mycobacterial survival under certain conditions (30). As such, while cytochrome *bc*_1_ is important for the growth of M. tuberculosis under laboratory conditions (25, 34–36), M. tuberculosis is able to survive during inhibition of cytochrome *bc*_1_ (37, 38), which is consistent with our data (Fig. 2B) and previously reported data indicating that QcrB inhibitors lack early bactericidal activity and are bacteriostatic for several weeks (19, 20, 31). Furthermore, it was reported that treatment of an M. tuberculosis mutant lacking cytochrome *bd* with QcrB inhibitors results in bacterial death (19, 31). Conversely, it was found that overexpression of cytochrome *bd* in M. tuberculosis can enable the bacteria to grow, despite cytochrome *bc*_1_ inhibition (8, 39). Together, these data indicate that cytochrome *bd* can compensate for cytochrome *bc*_1_ inhibition.

Expression of cytochrome *bc*_1_ and cytochrome *bd* has also been shown to change throughout the course of infection. In mice infected with M. tuberculosis, it was found that *qcrC* expression was the highest during acute infection (day 15) and that following 20 days of infection *qcrC* was transcriptionally downregulated 2- to 2.5-fold. In contrast, *cydA* was transcriptionally upregulated after 20 days, with the highest level of expression being detected at 30 days postinfection, when there was a 7-fold increase over the day 15 expression levels (40). These findings suggest that M. tuberculosis may rely more heavily on cytochrome *bc*_1_ during acute stages of infection, whereas cytochrome *bd* is activated at later stages of infection. These expression patterns may explain why it has been found that QcrB inhibitors work well to halt M. tuberculosis growth in the mouse when administered during the acute phase of infection (4, 12, 16, 19) but have a variable impact on the bacterial burden when treatment starts after the mice have been infected for more than 2 weeks, with most studies reporting modest decreases in the bacterial burden, if any decrease at all (4, 12, 19, 31). Some of the variability of QcrB inhibitor efficacy during the later stages of infection likely also depends on the timing of the treatment and the dosing of the compound.

Given the respiratory flexibilty in M. tuberculosis, interest has increased in using combinations of antibiotics that target different components of the ETC, a strategy that has been shown to lead to synergy in bactericidal effects (38, 41, 42). In particular, our studies with 4-amino-thieno[2,3-*d*]pyrimidines in the Δ*cydA* mutants imply that inhibition of QcrB would have an enhanced impact on M. tuberculosis in combination with inhibitors of cytochrome *bd*, as previously observed for Q203 (42). Together, these findings indicate that 4-amino-thieno[2,3-*d*]pyrimidines target QcrB and highlight the idea that the concurrent inhibition of both cytochrome *bc*_1_ and cytochrome *bd* is more effective than inhibition of cytochrome *bc*_1_ alone. In addition to potentiating other respiration inhibitors, a QcrB inhibitor has recently been shown to enhance pyrazinamide as well as rifampin monotherapy in mice, indicating that QcrB inhibitors may prove to be a useful addition to the current standard of care (12).

## MATERIALS AND METHODS

### Bacterial strains and growth conditions.

M. tuberculosis Erdman was cultured in Middlebrook 7H9 liquid medium supplemented with 60 μl/liter oleic acid, 5 g/liter bovine serum albumin, 2 g/liter dextrose, and 0.003 g/liter catalase (OADC) plus 0.5% glycerol and 0.05% Tween 80. For solid medium, Middlebrook 7H10 or 7H11 agar medium supplemented with OADC and 0.5% glycerol was used. M. smegmatis mc^2^155 was cultured in LB medium supplemented with 0.5% glycerol, 0.5% dextrose, and 0.05% Tween 80. Genetic deletion mutant strains of M. tuberculosis and M. smegmatis (Table 2) were generated using specialized transduction with the conditionally replicating phage phAE87 as previously described (43, 44). When appropriate, mycobacterial strains were selected on 20 μg/ml kanamycin and/or 50 μg/ml hygromycin.

**TABLE 2:** Strains and plasmids used in this study

Strain or plasmid	Description
Strains	
M. tuberculosis	
WT	Erdman
*qcrCAB*^WT^	Erdman Δ*qcrCAB*::Hyg^r^ *attB*::pMSG430-*qcrCAB_Mtb_*^WT^
*qcrCAB*^A178T^	Erdman Δ*qcrCAB*::Hyg^r^ *attB*::pMSG430-*qcrCAB_Mtb_*^A178T^
Δ*cydA*	Erdman Δ*cydA*::Hyg^r^ *attB*::pMSG430 (EV)
M. smegmatis	
WT	mc^2^155
csm208	mc^2^155 Δ*relA attB*::pMSG430-*relA_Mtb_*^53–394^
Δ*qcrCAB*	mc^2^155 Δ*qcrCAB*::Hyg^r^
Δ*cydA* + EV	mc^2^155 Δ*cydA*::Hyg^r^ *attB*::pMSG430 (EV)
Δ*cydA* + *cydAB*	mc^2^155 Δ*cydA*::Hyg^r^ *attB*::pMSG430-*cydAB_Msm_*
Plasmids	
pMSG430	Shuttle vector that harbors the L5 recombinase and *attP* site and a constitutive P*myc1*-*tetO* promoter, Kan^r^
pMSG430-*qcrCAB_Mtb_*^WT^	pMSG430 with *qcrCAB_Mtb_*^WT^ (P*myc1*-*tetO* promoter)
pMSG430-*qcrCAB_Mtb_*^A178T^	pMSG430 with *qcrCAB_Mtb_*^A178T^ (P*myc1*-*tetO* promoter)
pMSG430-*relA_Mtb_*^53–394^	pMSG430 with *relA_Mtb_*^53–394^ (P*myc1*-*tetO* promoter)
pMSG430-*cydAB_Msm_*	pMSG430 with *cydAB_Msm_* (P*myc1*-*tetO* promoter)
pMSG360	Cloning vector that harbors regions of homology to the phAE87 phage genome and multiple cloning sites upstream and downstream of an Hyg^r^ marker for engineering specialized transducing phage, Hyg^r^
pMSG360-*qcrCAB_Mtb_*	pMSG360 with homology to Erdman nucleotides 2448245–2448982 and 2452708–2453440
pMSG360-*cydA_Mtb_*	pMSG360 with homology to Erdman nucleotides 1809431–1810193 and 1807061–1808049
pMSG360-*qcrCAB_Msm_*	pMSG360 with homology to mc^2^155 nucleotides 4344291–4344965 and 4348476–4349188
pMSG360-*cydA_Msm_*	pMSG360 with homology to mc^2^155 nucleotides 3317608–3318615 and 3315796–3316290

### Initial screen.

Logarithmically growing M. smegmatis strain csm208 was inoculated into 96-well dishes containing 100 μM compounds in 200 μl LB at a starting optical density at 600 nm (ODλ_600_) of 0.2 and incubated with shaking at 37°C in a Tecan M200 Pro plate reader, with ODλ_600_ measurements being taken in each well every 15 s.

### Microplate alamarBlue assays (MABAs).

Logarithmically growing M. tuberculosis was inoculated into 7H9 medium in 96-well plates with wells containing increasing concentrations of compound. M. tuberculosis was inoculated at an ODλ_600_ of 0.0008, corresponding to approximately 4 × 10^5^ CFU/ml in 200 μl per well. The plates were incubated at 37°C in 5% CO_2_ for 1 week, at which point 32.5 μl of a mixture containing an 8:5 ratio of 0.6 mM resazurin (Sigma) dissolved in 1× phosphate-buffered saline to 20% Tween 80 was added, and the production of fluorescent resorufin was measured after incubation at 37°C in 5% CO_2_ overnight. For M. tuberculosis, samples were removed from the plate and mixed with formalin to kill the M. tuberculosis bacteria before measuring the fluorescence. For M. smegmatis, the assay plate was measured directly. Fluorescence was measured on a Tecan M200 Pro plate reader with an excitation λ of 530 nm and an emission λ of 590 nm. For each assay, medium alone served as a negative control, and untreated M. tuberculosis or M. smegmatis was included as a positive control. The percent inhibition was calculated as the {[(fluorescence of the positive control − fluorescence of the negative control) − (fluorescence of the sample − fluorescence of the negative control)]/(fluorescence of the positive control − fluorescence of the negative control)} × 100.

### Selection and sequencing of resistant mutants.

Resistant mutants were selected on 7H11 agar medium containing 10 μM CWHM-728. Approximately 2.5 × 10^8^ CFU of WT M. tuberculosis Erdman was spread per plate, and the plate was incubated at 37°C for 12 weeks. Genomic DNA was isolated using cetyltrimethylammonium bromide-lysozyme lysis, followed by chloroform-isoamyl alcohol extraction and isopropanol precipitation, as previously described (45). Whole-genome sequencing was performed by use of an Illumina HiSeq sequencer with 50-bp single-end reads. The identification of single nucleotide polymorphisms (SNPs) was done using SeqMan NGen software (DNASTAR). The genomes were assembled and compared to the genomic DNA from the WT parental control strain to identify SNPs that may be responsible for resistance to CWHM-728. The mutations identified in *qcrB* by whole-genome sequencing were subsequently confirmed by Sanger sequencing. For Sanger sequencing of the *qcrB* locus, the genomic region was PCR amplified using primers erdqcrBfwxbaI430 (GTCTAGAATGAGTCCGAAACTGAGTCCGCC) and RK27 (GAAGCTTTCGCCGGGCTAGTGCTCGCCGTC) and then sequenced using the same two primers (GeneWiz).

### Computational modeling.

A homology model of M. tuberculosis QcrB was built using the crystal structure of Rhodobacter sphaeroides QcrB (PDB accession number 2QJP) (28) as the template structure, using the sequence alignment from Ko and Choi (29). The model, which was built in the Molecular Operating Environment (MOE 2016.08; Chemical Computing Group, Montreal, Quebec, Canada), was placed into a membrane environment (46), and energy was minimized. The geometry of the [2Fe-2S] center and the disulfide bond pattern adjacent to the inhibitor-binding site were assumed to match those of the template structure. CWHM-728 was docked into the homology model using the GOLD (v5.5) program (47).

### ATP measurements.

M. tuberculosis or M. smegmatis was inoculated into 7H9 medium with or without the test compounds at an ODλ_600_ of 0.1 and incubated with shaking at 37°C for 24 h (M. tuberculosis) or 12 h (M. smegmatis). An aliquot of the culture was heat inactivated at 95°C for 20 min and diluted 1:100. Diluted samples were mixed with the BacTiter-Glo assay (Promega) reagent at a 1:1 ratio, and luminescence was quantified on a Tecan M200 Pro plate reader (integration = 1 s). Relative luminescence units (RLU) were normalized to the number of log_10_ CFU in the sample to account for differences in bacterial number.

### Compounds.

CB37, CWHM-728 (CB81), CWHM-935, CWHM-936, CWHM-941, CWHM-950, CWHM-937, CWHM-946, CWHM-951, and CWHM-942 were purchased from ChemBridge Corporation. Synthesis of the compounds CWHM-1069, CWHM-1020, CWHM-1022, CWHM-1021, CWHM-1304, CWHM-1303, CWHM-1306, and CWHM-1023 is described in Text S1 in the supplemental material, and liquid chromatography-mass spectrometry (LC-MS), ^1^H nuclear magnetic resonance (NMR), and ^13^C NMR analyses were done on CWHM-1023 to confirm the purity and identity of the synthesized compound (Fig. S3 and S4). Q203 was acquired from Enamine (catalog number EN-300-218150), and both bedaquiline (catalog number 465749185) and thioridazine (catalog number 1662504) were purchased from Sigma-Aldrich; all three compounds were tested in the MABA for comparative purposes.

10.1128/mSphere.00606-19.1 TEXT S1 Supplemental information. Download Text S1, PDF file, 0.3 MB. Copyright © 2019 Harrison et al. 2019 Harrison et al. This content is distributed under the terms of the Creative Commons Attribution 4.0 International license.

10.1128/mSphere.00606-19.4 FIG S3 LC-MS spectra for CWHM-1023. CWHM-1023 was subjected to LC-MS analysis as described in Text S1 in the supplemental material. Download FIG S3, TIF file, 0.6 MB. Copyright © 2019 Harrison et al. 2019 Harrison et al. This content is distributed under the terms of the Creative Commons Attribution 4.0 International license.

10.1128/mSphere.00606-19.5 FIG S4 NMR spectra for CWHM-1023. CWHM-1023 was subjected to ^1^H NMR (A) and ^13^C NMR (B) analysis. The inset in panel A depicts the chemical structure of CWHM-1023. Download FIG S4, TIF file, 0.4 MB. Copyright © 2019 Harrison et al. 2019 Harrison et al. This content is distributed under the terms of the Creative Commons Attribution 4.0 International license.

### Data availability.

The sequence data are publicly available in the NCBI Sequence Read Archive under accession no. PRJNA561987.
