# Supplementary figures and images for: Identification of 4-Amino-Thieno[2,3-d]Pyrimidines as QcrB Inhibitors in Mycobacterium tuberculosis
Source: mSphere. 2019 Sep 11;4(5):e00606-19. doi: 10.1128/mSphere.00606-19 (PMC6739496; doi:10.1128/mSphere.00606-19)

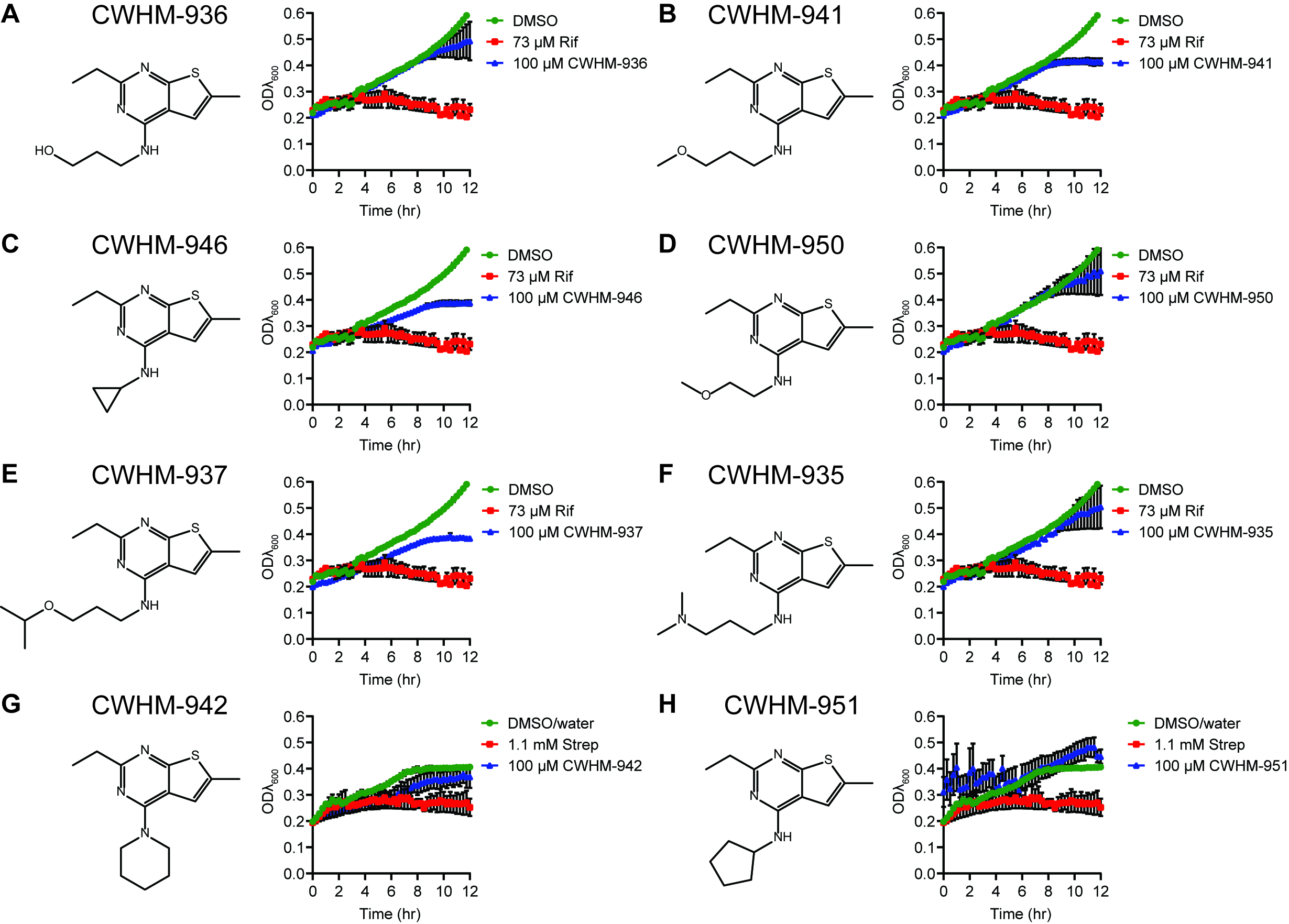

Supplement: FIG S1 [file mSphere.00606-19-sf001.tif]

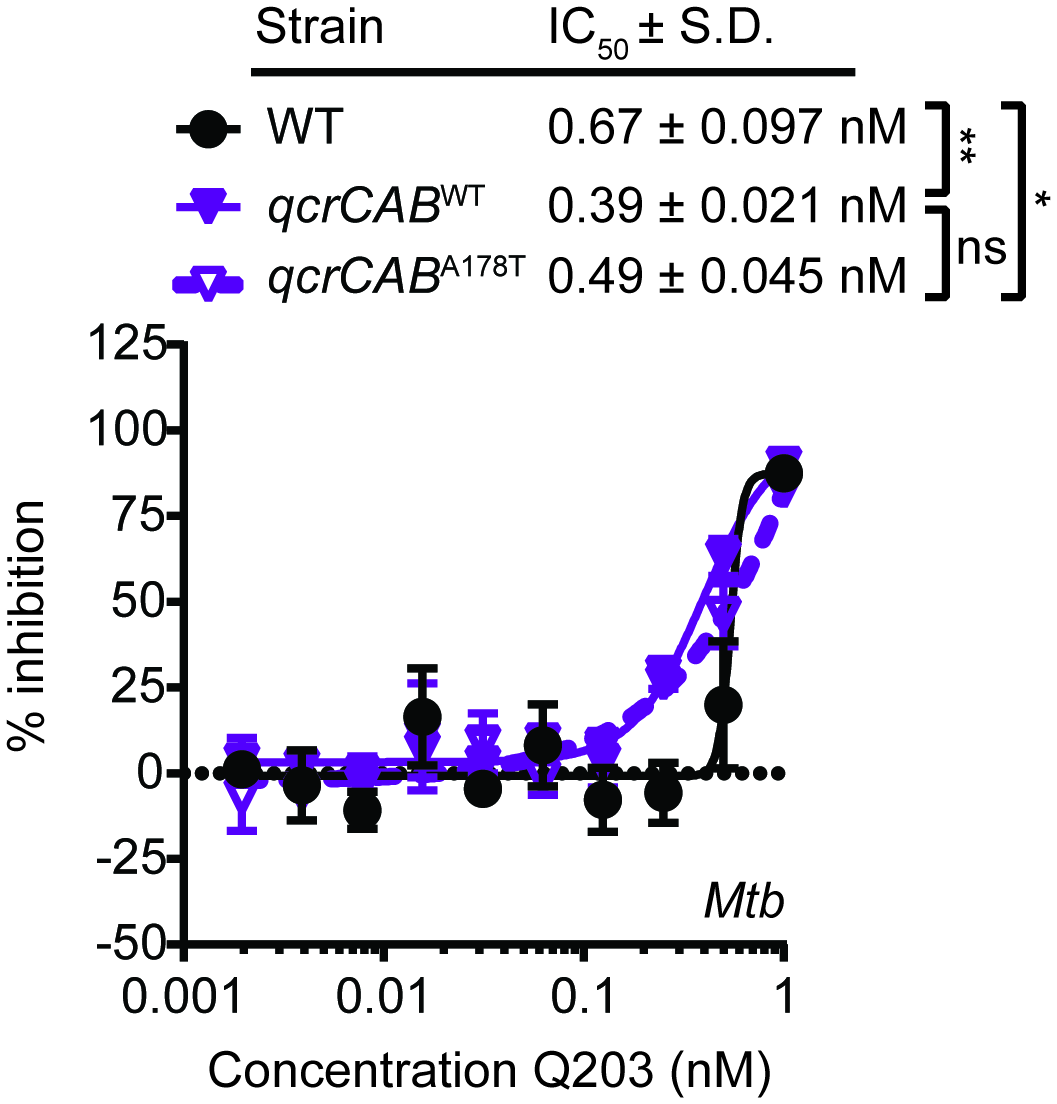

Supplement: FIG S2 [file mSphere.00606-19-sf002.tif]

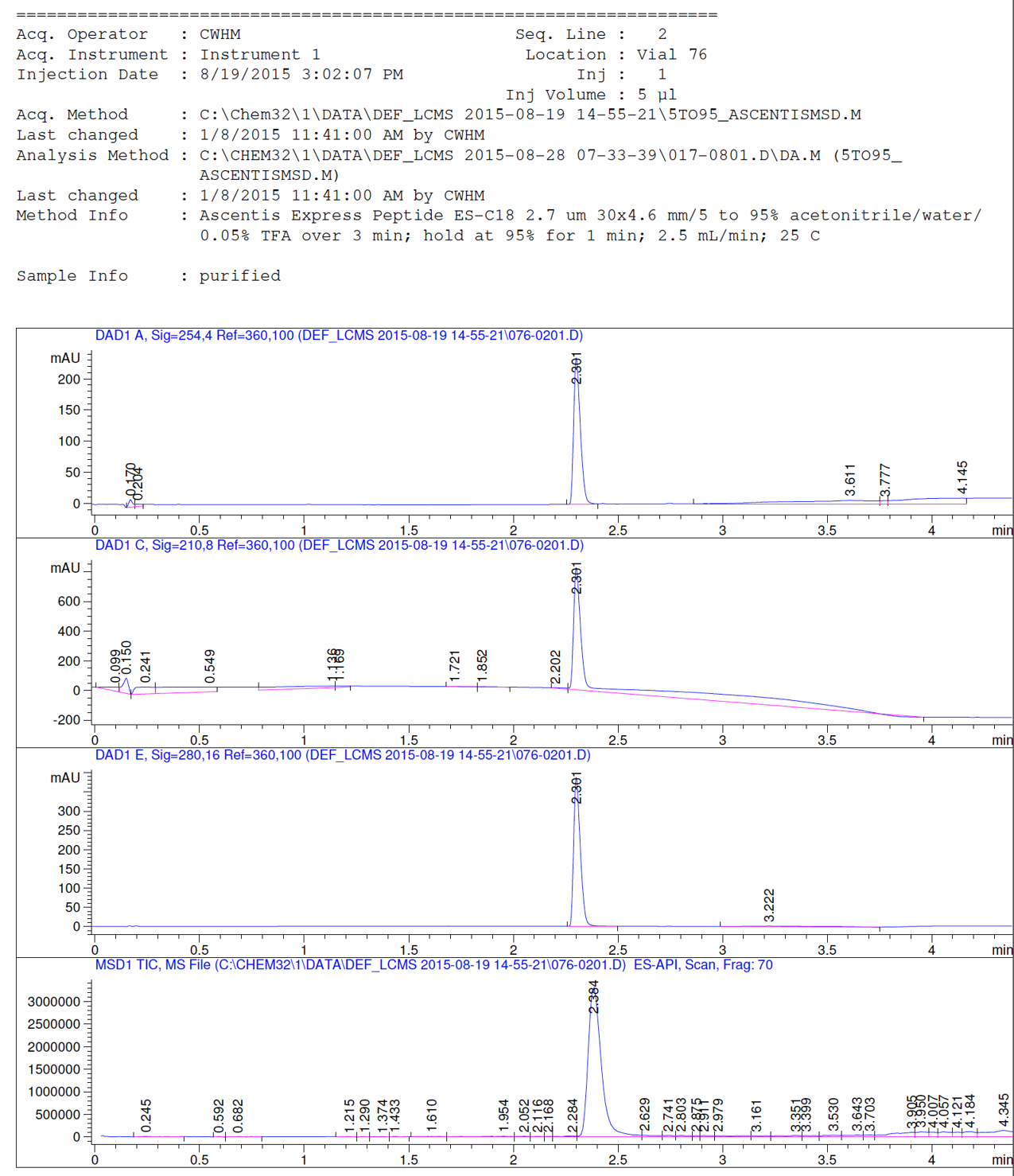

Supplement: FIG S3 [file mSphere.00606-19-sf003.tif]

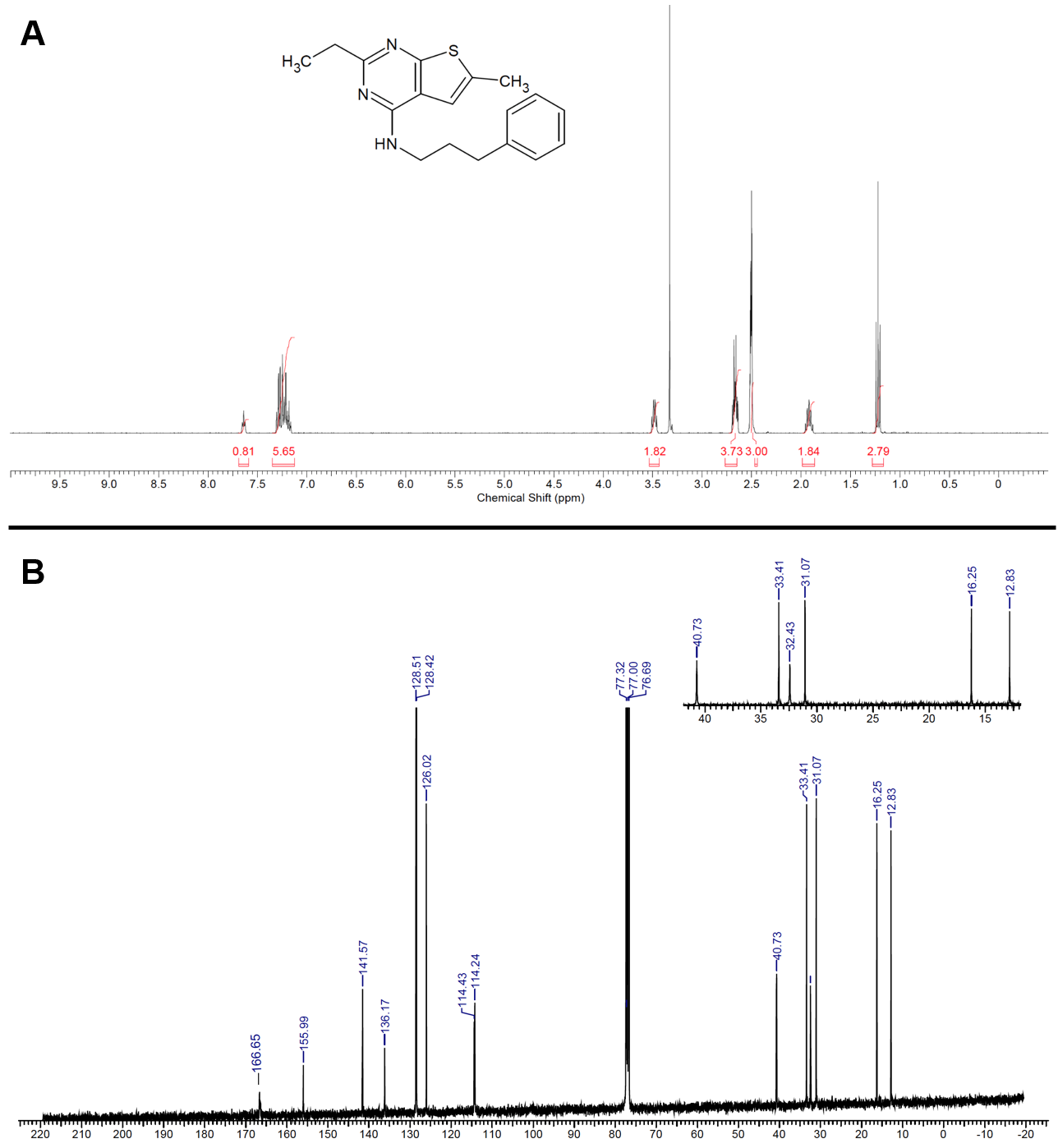

Supplement: FIG S4 [file mSphere.00606-19-sf004.tif]
