# Supplementary material for: Identification of 4-Amino-Thieno[2,3-d]Pyrimidines as QcrB Inhibitors in Mycobacterium tuberculosis
Source: mSphere. 2019 Sep 11;4(5):e00606-19. doi: 10.1128/mSphere.00606-19 (PMC6739496; doi:10.1128/mSphere.00606-19)
Supplement: TABLE S1 [file mSphere.00606-19-st001.docx]

| **Entry** | **Compound Number** | **Source** | **R** | **MABA in *Mtb* IC_50_ (µM)** | **clogP*^a^*** |
| --- | --- | --- | --- | --- | --- |
| 1 | CB37 | ChemBridge 9204833 | __ | >50 | 3.2 |
| 2 | CWHM-728 (CB81) | ChemBridge 9258457; resynthesis |  | 2.7*^b^* | 4.5 |
| 3 | CWHM-935 | ChemBridge 9219957 |  | >50 | 3.4 |
| 4 | CWHM-936 | ChemBridge 9220073 |  | 26 | 2.7 |
| 5 | CWHM-941 | ChemBridge 9245116 |  | 7.8 | 3.2 |
| 6 | CWHM-950 | ChemBridge 9277737 |  | 17 | 3.2 |
| 7 | CWHM-937 | ChemBridge 9225952 |  | 0.32 | 4.0 |
| 8 | CWHM-946 | ChemBridge 9264302 |  | 0.62 | 3.7 |
| 9 | CWHM-951 | ChemBridge 9280331 |  | >50 | 4.6 |
| 10 | CWHM-942 | ChemBridge 9252275 |  | 49 | 4.7 |
