# Supplementary material for: Identification of 4-Amino-Thieno[2,3-d]Pyrimidines as QcrB Inhibitors in Mycobacterium tuberculosis
Source: mSphere. 2019 Sep 11;4(5):e00606-19. doi: 10.1128/mSphere.00606-19 (PMC6739496; doi:10.1128/mSphere.00606-19)
Supplement: TABLE S2 [file mSphere.00606-19-st002.docx]

| **Entry** | **Compound Number** | **Source** | **R** | **MABA in *Mtb* IC_50_ (µM)** | **clogP*^a^*** |
| --- | --- | --- | --- | --- | --- |

| 1 | CWHM-728 (CB81) | ChemBridge 9258457; resynthesis |  | 3.2*^b^* | 4.5 |
| --- | --- | --- | --- | --- | --- |
| 2 | CWHM-1069 | synthesis |  | 30 | 4.2 |
| 3 | CWHM-1020 | synthesis |  | 0.11 | 4.6 |
| 4 | CWHM-1022 | synthesis |  | 0.32 | 4.8 |
| 5 | CWHM-1021 | synthesis |  | 3.8 | 5.2 |
| 6 | CWHM-1304 | synthesis |  | 25 | 5.0 |
| 7 | CWHM-1303 | synthesis |  | 11 | 5.0 |
| 8 | CWHM-1306 | synthesis |  | 0.15 | 5.3 |
| 9 | CWHM-1023 | synthesis |  | 0.083*^b^* | 5.7 |
| 10 | Q203 | Enamine EN-300-218150 | N/A | 0.0015 | 6.7 |
| 11 | Bedaquiline | Sigma 465749185 | N/A | <0.078 | 7.3 |
| 12 | Thioridazine | Sigma 1662504 | N/A | 11.2 | 5.9 |
