# Supplementary material for: Identification of 4-Amino-Thieno[2,3-d]Pyrimidines as QcrB Inhibitors in Mycobacterium tuberculosis
Source: mSphere. 2019 Sep 11;4(5):e00606-19. doi: 10.1128/mSphere.00606-19 (PMC6739496; doi:10.1128/mSphere.00606-19)
Supplement: TEXT S1 [file mSphere.00606-19-s0001.pdf]

Supporting Information

Chemical Synthesis: Instrumentation and General Methods. The starting materials were obtained from commercial sources and used without further purification after verifying their purities by LC-MS analysis. Reactions were performed under an atmosphere of dry nitrogen unless otherwise stated. Solvents were analytical grade and used as supplied. Analytical HPLC analyses were performed on an Agilent 1100 system and LC-MS analyses were conducted on Agilent 1100 Series LC/MSD (G1946C) single quadrupole mass spectrometer system equipped with an electrospray ionization (ESI) source. Reverse-phase preparative HPLC purifications were performed either on a Biotage SP4 HPFC system or on a CombiFlash*Rf* (Teledyne Isco) system using a variable dual wavelength UV detector on a Biotage KP-C18-HS 120 g SNAP column and on Redisep Rf Gold C18 cartridges using acetonitrile/water gradient containing 0.05% TFA. Normal phase preparative HPLC purifications were performed either on a Biotage SP4 HPFC system or on a CombiFlash*Rf* (Teledyne Isco) system using a variable dual wavelength UV detector with pre-packed Biotage KP-SIL SNAP cartridges and Redisep Rf silica gel (Isco) cartridges and ethyl acetate/hexanes gradients.

All final compounds were analyzed by analytical HPLC using a C18 analytical column with a diode array detector and peaks were monitored at 210, 254 and 280 nM for their purity. ^{1}H and ^{19}F NMR spectra were recorded in deuterated solvents (DMSO-d_6, CD_3OD and $CDCl_3$) on a Bruker *Avance*-III/400 MHz spectrometer equipped with a Broad Band NMR probe. The signal of the deuterated solvent was used as an internal reference. The chemical shifts are expressed in ppm (δ) and coupling constants (*J*) are reported in hertz (Hz). Samples were analyzed by high resolution mass spectrometry

using flow injection analysis coupled to a Q-Exactive mass spectrometer (Thermo Fisher Scientific, Waltham, MA USA). Samples were injected at 50µl/min with 90% ACN, 10% water with 0.1% formic acid. The electrospray voltage was set to 1,500 V and the accuracy of the instrument was determined to be less than 5 ppm.

Synthesis of thieno[2,3-*d*]pyrimidine analogs. The general procedures described by Prasad and coworkers (1) were modified as described below.

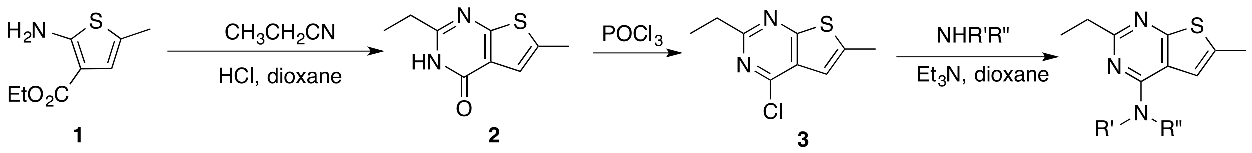

2-Ethyl-6-methyl-3H-thieno[2,3-*d*]pyrimidin-4-one (2). A dark red mixture of 2-amino-3-ethoxycarbonyl-5-methylthiophene (**1**; 648.0 mg, 3.5 mmol) and propionitrile (0.5 mL, 7.0 mmol) was treated with 4.0 M HCl in 1,4-dioxane (3 mL, 12.0 mmol) at room temperature. The reaction mixture quickly turned to a thick yellow-orange paste. An additional 2 mL 4N HCl in 1,4-dioxane was added after 30 min and the reaction mixture was heated at 50 °C to give a red solution. LC-MS analysis of the reaction mixture after 1.5 h showed the uncyclized intermediate product and the intermediate's mass: *m/z* 241 $[M+H]^+$; no traces of the starting materials were present. After heating at 50 °C for 2 h, the reaction mixture was heated at 110 °C to give a dark red solution. A thick yellow paste began to form within 1 h. The reaction mixture was heated at 110 °C overnight. The solvent was evaporated *in vacuo* to afford a yellow-brown solid. The solid was dissolved in acetonitrile (20 mL) and cooled to room temperature to afford a crystalline

precipitate. The solid was filtered, washed with acetonitrile (2×10 mL) and dried *in vacuo* to give a cream crystalline solid (648.0 mg, yield 95%). LC-MS purity >98%; *m/z* 195 $[M+H]^+$ and 217 $[M+Na]^+$. ^{1}H NMR (400 MHz, DMSO-d_6): δ 1.20 (t, *J* = 7.58 Hz, 3H), 2.47 (d, *J* = 1.22 Hz, 3H), 2.61 (q, *J* =7.58 Hz, 2H), 7.02 (d, *J* = 1.22 Hz, 1H), 11.59 (br. s, 1H).

4-Chloro-2-ethyl-6-methylthieno[2,3-*d*]pyrimidine (3). A suspension of 2-ethyl-6-methyl-3H-thieno[2,3-*d*]pyrimidin-4-one (648.0 mg, 3.34 mmol) in phosphorus oxychloride (4 mL, 42.91 mmol)) was heated at refluxing conditions. Within 1 h a light brown-orange solution was obtained. The solvent was evaporated *in vacuo* to afford a light orange-brown viscous liquid. The liquid was poured onto crushed ice-water to give a cream precipitate. The mixture was neutralized with a saturated $NaHCO_3$ solution to give a cream precipitate. The precipitate was extracted with ethyl acetate (2×25 mL), the aqueous and the organic layers were separated, the organic layer was washed with brine (1×25 mL) and dried over anhydrous Na2SO4, filtered and evaporated *in vacuo* to afford a very light brown liquid which solidified to a cream crystalline solid (620.0 mg, yield 88%). LC-MS purity >98%; *m/z* 213 $[^{35Cl}M+H]^+$ and 215 $[^{37Cl}M+H]^+$. ^{1}H NMR (400 MHz, DMSO-d_6): δ 1.30 (t, *J* = 7.55 Hz, 3H), 2.63 (d, *J* = 1.34 Hz, 3H), 2.96 (q, *J* = 7.54 Hz, 2H), 7.23 (d, *J* = 1.34 Hz, 1H).

2-Ethyl-6-methyl-*N*-(4,4,4-trifluorobutyl)thieno[2,3-d]pyrimidin-4-amine (CWHM-1020). A solution of 4-chloro-2-ethyl-6-methyl-thieno[2,3-*d*]pyrimidine (50 mg, 0.235 mmol), DIEA (50 µL, 0.292 mmol) and 4,4,4-trifluorobutylamine (66 µL, 0.705 mmol) in 1,4-dioxane was heated to 140 °C for 60 min in a microwave reactor. The reaction mixture was partitioned between water and DCM. The DCM layer was separated and

concentrated and the crude product was purified by reverse phase HPLC to give the desired product. The residue was dissolved in acetonitrile containing a couple drops of methanol and eluted through a SiliaPrep Carbonate 6mL-1g plug. Evaporation of the solvent *in vacuo* afforded the product as a white solid (26.1 mg, yield 37%). LC-MS purity >98%; *m/z* 304 $[M + H]^+$. ^{1}H NMR (400 MHz, DMSO-d_6): δ 7.68 (t, *J* = 5.53 Hz, 1H), 7.18 (d, *J* = 1.34 Hz, 1H), 3.53 (q, *J* = 6.66 Hz, 2H), 2.67 (q, *J* = 7.56 Hz, 2H), 2.49 (d, *J* = 1.00 Hz, 3H), 2.24 - 2.44 (m, 2H), 1.76 - 1.90 (m, 2H), 1.22 (t, *J* = 7.55 Hz, 3H); ^{19}F NMR (376 MHz, DMSO-d_6): δ -64.68. HRMS (ESI) m/z: $[M + H]^+$ Calcd for $C_{13}H_{17}F_3N_3S$ 304.1090; found 304.1078.

***N*-Butyl-2-ethyl-N,6-dimethylthieno[2,3-d]pyrimidin-4-amine (CWHM-1021).** A solution of 4-chloro-2-ethyl-6-methyl-thieno[2,3-*d*]pyrimidine (50 mg, 0.235 mmol), DIEA (50 µL, 0.292 mmol) and *N*-methyl-*n*-butylamine (84 µL, 0.705 mmol) in 1,4-dioxane was heated to 140 °C for 60 min in a microwave reactor. The reaction mixture was partitioned between water and DCM. The DCM layer was separated and concentrated and the crude product was purified by reverse phase HPLC to give the desired product. The residue was dissolved in acetonitrile containing a couple drops of methanol and eluted through a SiliaPrep Carbonate 6mL-1g plug to neutralize. Evaporation of the solvent *in vacuo* afforded as an oil which was dissolved in acetonitrile/water and lyophilized to give a white solid (24.8 mg, yield 40%). LC-MS purity >98%; *m/z* 264 $[M + H]^+$. ^{1}H NMR (400 MHz, DMSO-d_6): δ 7.24 (d, *J* = 1.28 Hz, 1H), 3.66 - 3.77 (m, 2H), 3.32 (s, 3H), 2.66 (q, *J* = 7.58 Hz, 2H), 2.49 (d, *J* = 1.16 Hz, 3H), 1.55 - 1.65 (m, 2H), 1.27 - 1.39 (m, 2H), 1.23 (t, *J* = 7.58 Hz, 3H), 0.92 (t, *J* = 7.37 Hz, 3H). HRMS (ESI) m/z: $[M + H]^+$ Calcd for $C_{14}H_{22}N_3S$ 264.1529; found 264.1519.

2-Ethyl-6-methyl-*N*-(3-methylbutyl)thieno[2,3-*d*]pyrimidin-4-amine (CWHM-1022). A solution of 4-chloro-2-ethyl-6-methyl-thieno[2,3-*d*]pyrimidine (50 mg, 0.235 mmol), DIEA (50 µL, 0.292 mmol) and isopentylamine (82 µL, 0.705 mmol) in 1,4-dioxane was heated to 140 °C for 60 min in a microwave reactor. The reaction mixture was partitioned between water and DCM. The DCM layer was separated and concentrated and the crude product was purified by reverse phase HPLC to give the desired product. The residue was dissolved in acetonitrile containing a couple drops of methanol and eluted through a SiliaPrep Carbonate 6mL-1g plug to neutralize TFA. Evaporation of the solvent *in vacuo* afforded the product as a white solid (29.0 mg, yield 47%). LC-MS purity >98%; *m/z* 264 $[M + H]^+$. ^{1}H NMR (400 MHz, DMSO-d_6): δ 7.54 (t, *J* = 5.44 Hz, 1H), 7.19 (d, *J* = 1.22 Hz, 1H), 3.42 - 3.55 (m, 2H), 2.66 (q, *J* = 7.58 Hz, 2H), 2.48 (s, 3H), 1.63 (td, *J* = 6.65, 13.36 Hz, 1H), 1.48 (q, *J* = 6.85 Hz, 2H), 1.22 (t, *J* = 7.58 Hz, 3H), 0.92 (d, *J* = 6.54 Hz, 6H). HRMS (ESI) m/z: $[M + H]^+$ Calcd for $C_{14}H_{22}N_3S$ 264.1529; found 264.1518.

2-Ethyl-6-methyl-*N*-(3-phenylpropyl)thieno[2,3-*d*]pyrimidin-4-amine (CWHM-1023). A solution of 4-chloro-2-ethyl-6-methylthieno[2,3-*d*]pyrimidine (50 mg, 0.235 mmol), DIEA (50 µL, 0.292 mmol) and 3-phenyl-*n*-propylamine (100 µL, 0.705 mmol) in 1,4-dioxane was heated to 140 °C for 60 min in a microwave reactor. The reaction mixture was partitioned between water and DCM. The DCM layer was separated and concentrated and the crude product was purified by reverse phase HPLC to give the desired product. The residue was dissolved in acetonitrile containing a couple drops of methanol and eluted through a SiliaPrep Carbonate 6mL-1g plug to neutralize TFA. Evaporation of the solvent *in vacuo* afforded the product as a white solid (32.1 mg, yield

44%). LC-MS purity >98%; *m/z* 312 $[M + H]^+$ (Fig. S3). ^1H NMR (400 MHz, DMSO-d_6): δ 7.64 (t, *J* = 5.47 Hz, 1H), 7.12 - 7.35 (m, 6H), 3.44 - 3.54 (m, 2H), 2.67 (qd, *J* = 3.79, 11.37 Hz, 4H), 2.50 (d, *J* = 1.16 Hz, 3H), 1.86 - 1.99 (m, 2H), 1.22 (t, *J* = 7.58 Hz, 3H) (Fig. S4A). ^{13}C NMR (100 MHz, $CDCl_3$) δ 166.7, 156.0, 141.6, 136.2, 128.5, 128.4, 126.0, 114.4, 114.2, 40.7, 33.4, 32.1, 16.3, 12.8 (Fig. S4B). HRMS (ESI) m/z: $[M + H]^+$ Calcd for $C_{18}H_{22}N_3S$ 312.1529; found 312.1517.

***N*-tert-Butyl-2-ethyl-6-methyl-thieno[2,3-*d*]pyrimidin-4-amine (CWHM-1069).** A solution of 4-chloro-2-ethyl-6-methyl-thieno[2,3-*d*]pyrimidine (60.5 mg, 0.28 mmol), DIEA (100 µL, 0.58 mmol) and *tert*-butylamine (90 µL, 0.85 mmol) in 1,4-dioxane (1 mL) was heated at from 140 to 200 °C in a microwave reactor for several hours (>18 h) to give a yellow-orange solution. The solvent was evaporated *in vacuo* to give a light tan crystalline solid and the crude residue was purified by reverse-phase preparative HPLC to afford a colorless to cream crystalline residue. The purified residue was dissolved in acetonitrile containing a trace of methanol and the solution was passed through a Silia*Prep* Carbonate (Si-CO_3) 6 mL-1 g cartridge. The filtrate was evaporated *in vacuo* to afford a colorless powder (37.3 mg, yield 53%). LC-MS purity >98%; *m/z* 250 $[M + H]^+$. ^1H NMR (400 MHz, DMSO-d_6): δ 1.24 (t, *J* = 7.58 Hz, 3H), 1.50 (s, 9H), 2.48 (d, *J* = 1.22 Hz, 3H), 2.68 (q, *J* = 7.58 Hz, 2H), 6.82 (s, 1H), 7.37 (d, *J* = 1.22 Hz, 1H). HRMS (ESI) m/z: $[M + H]^+$ Calcd for $C_{13}H_{20}N_3S$ 250.1372; found 250.1362.

***N*-Benzyl-2-ethyl-6-methylthieno[2,3-*d*]pyrimidin-4-amine (CWHM-1303).** A solution of 4-chloro-2-ethyl-6-methyl-thieno[2,3-*d*]pyrimidine (67.0 mg, 0.315 mmol), DIEA (110 µL, 0.643 mmol) and benzylamine (105 µL, 0.96 mmol) in 1,4-dioxane (2 mL) was heated at 140 °C in a microwave reactor for 3 h to give a pale yellow solution. The

solvent was evaporated *in vacuo* to give a cream crystalline solid. The crude residue was purified by reverse-phase preparative HPLC to afford a colorless viscous liquid containing a colorless crystalline solid (130.3 mg). The purified residue was dissolved in acetonitrile containing a trace of methanol and the solution was passed through a Silia*Prep* Carbonate (Si-CO_3) 6 mL-1 g cartridge to neutralize TFA. The filtrate was evaporated *in vacuo* to afford a colorless crystalline solid (90.0 mg, yield 100%). LC-MS purity >98%; *m/z* 284 $[M + H]^+$. ^1H NMR (400 MHz, $CDCl_3$): δ 1.37 (t, *J* = 7.58 Hz, 3H), 2.53 (s, 3H), 2.87 (q, *J* = 7.58 Hz, 2H), 4.84 (d, *J* = 5.62 Hz, 2H), 5.17 (br. s, 1H), 6.71 (s, 1H), 7.28-7.37 (m, 3H), 7.38-7.42 (m, 2H). HRMS (ESI) m/z: $[M + H]^+$ Calcd for $C_{16}H_{18}N_3S$ 284.1216; found 284.1205.

2-Ethyl-6-methyl-*N*-phenylthieno[2,3-*d*]pyrimidin-4-amine (CWHM-1304). A solution of 4-chloro-2-ethyl-6-methyl-thieno[2,3-*d*]pyrimidine (71.6 mg, 0.34 mmol), DIEA (120 μL, 0.70 mmol) and aniline (95 μL, 1.04 mmol) in 1,4-dioxane (2 mL) was heated at 200 °C in a microwave reactor for 6 h to give a dark yellow solution. The solvent was evaporated *in vacuo* to give a pale yellow viscous liquid solidified slowly to a dirty cream crystalline solid (176.6 mg). The crude residue was purified by reverse-phase preparative HPLC to afford a colorless crystalline precipitate in water. The solid was filtered, washed with water (3×10 mL) and dried *in vacuo* to afford a colorless solid. The purified residue was dissolved in acetonitrile containing a trace of methanol and the solution was passed through a Silia*Prep* Carbonate (Si-CO_3) 6 mL-1 g cartridge to neutralize TFA. The filtrate was evaporated *in vacuo* to afford a cream crystalline solid (88.9 mg; yield 98%). LC-MS purity >98%; *m/z* 270 $[M + H]^+$. ^1H NMR (400 MHz, $CDCl_3$): δ 1.41 (t, *J* = 7.58 Hz, 3H), 2.56 (s, 3H), 2.94 (q, *J* = 7.58 Hz, 2H), 6.73 (s, 1H),

6.80 (br. s, 1H), 7.14 (t, *J* = 7.45 Hz, 1H), 7.39 (t, *J* = 7.70 Hz, 2H), 7.71 (d, *J* = 8.31 Hz, 2H). HRMS (ESI) m/z: $[M + H]^+$ Calcd for $C_{15}H_{16}N_3S$ 270.1059; found 270.1048.

2-Ethyl-6-methyl-*N*-phenethylthieno[2,3-*d*]pyrimidin-4-amine (CWHM-1306). A solution of 4-chloro-2-ethyl-6-methyl-thieno[2,3-*d*]pyrimidine (67.0 mg, 0.32 mmol), DIEA (110 µL, 0.64 mmol) and 2-phenylethylamine (119 µL, 0.95 mmol) in 1,4-dioxane (2 mL) was heated at 140 °C in a microwave reactor for 3 h to give a colorless solution and the solvent was evaporated *in vacuo* to give a pale viscous liquid. The crude residue was purified by reverse-phase preparative HPLC to afford a colorless crystalline solid (132.0 mg). The purified residue was dissolved in acetonitrile containing a trace of methanol and the solution was passed through a Silia*Prep* Carbonate (Si-CO3) 6 mL-1 g cartridge. The filtrate was evaporated *in vacuo* to afford a colorless crystalline solid (90.0 mg, yield 96%). LC-MS purity >98%; *m/z* 298 $[M + H]^+$. ^1H NMR (400 MHz, $CDCl_3$): δ 1.39 (t, *J* = 7.58 Hz, 3H), 2.53 (s, 3H), 2.87 (q, *J* = 7.58 Hz, 2H), 2.99 (t, *J* = 6.97 Hz, 2H), 3.89 (q, *J* = 6.60 Hz, 2H), 4.95 (br. s, 1H), 6.60 (s, 1H), 7.23-7.29 (m, 3H), 7.32-7.39 (m, 2H). HRMS (ESI) m/z: $[M + H]^+$ Calcd for $C_{17}H_{20}N_3S$ 298.1372; found 298.1361.

References:

1. Prasad MR, Rao ARR, Rao PS, Rajan KS. 2001. Microwave-Assisted Synthesis of Novel 5-Substituted-2,3-dihydroimidazo[1,2-c]thieno[3,2-e]pyrimidines. Synthesis (Stuttg) 2001:2119–2123.
